# Application of plasma metagenomic next-generation sequencing improves prognosis in hematology patients with neutropenia or hematopoietic stem cell transplantation for infection

**DOI:** 10.3389/fcimb.2024.1338307

**Published:** 2024-02-02

**Authors:** Yuhui Chen, Jinjin Wang, Xinai Gan, Meng Li, Yi Liao, Yongzhao Zhou, Ting Niu

**Affiliations:** ^1^ Department of Hematology, West China Hospital, Sichuan University, Chengdu, China; ^2^ Integrated Care Management Center, West China Hospital, Sichuan University, Chengdu, China

**Keywords:** metagenomic next-generation sequencing, infection, hematology patients, diagnosis, prognosis

## Abstract

**Introduction:**

Metagenomic next-generation sequencing (mNGS) is a novel technique for detecting pathogens. This retrospective study evaluated the diagnostic value of mNGS using plasma for infections in hematology patients and its impact on clinical treatment and prognosis in different subgroups of hematology patients.

**Methods:**

A total of 153 hematology patients with suspected infection who underwent mNGS using plasma were enrolled in the study. Their clinical histories, conventional microbiological test (CMT) results, mNGS results, treatment and prognosis were retrospectively analyzed.

**Results:**

In 153 plasma samples, mNGS yielded a higher positivity rate than CMT (total: 88.24% vs. 40.52%, P<0.001; bacteria: 35.95% vs. 21.57%, P < 0.01; virus: 69.93% vs. 21.57%, P<0.001; fungi: 20.26% vs. 7.84%, P<0.01). mNGS had a higher positivity rate for bacteria and fungi in the neutropenia group than in the non-neutropenia group (bacteria: 48.61% vs. 24.69%, P<0.01; fungi: 27.78% vs. 13.58%, P<0.05). mNGS demonstrated a greater advantage in the group of patients with hematopoietic stem cell transplantation (HSCT). Both the 3-day and 7-day efficacy rates in the HSCT group were higher than those in the non-HSCT group (3-day: 82.22% vs. 58.65%, P < 0.01; 7-day: 88.89% vs. 67.31%, P < 0.01), and the 28-day mortality rate was lower in the HSCT group than in the non-HSCT group (6.67% vs. 38.89%, P < 0.000). The neutropenia group achieved similar efficacy and mortality rates to the non-neutropenia group (7-day efficiency rate: 76.39% vs. 71.43%, P > 0.05; mortality rate: 29.17% vs. 29.63%, P > 0.05) with more aggressive antibiotic adjustments (45.83% vs. 22.22%, P < 0.01).

**Conclusion:**

mNGS can detect more microorganisms with higher positive rates, especially in patients with neutropenia. mNGS had better clinical value in patients with hematopoietic stem cell transplantation (HSCT) or neutropenia, which had a positive effect on treatment and prognosis.

## 1 Introduction

Due to the frequent use of chemotherapy, corticosteroids and immunosuppressants in hematology patients, which leads to neutropenia and immunosuppression, hematology patients are susceptible to invasion by a variety of pathogens (Rolston et al., 2007). Infection is one of the leading causes of death in hematology patients, especially with neutropenia or hematopoietic stem cell transplantation (HSCT). It was reported that bloodstream infections occur in 13% to 60% of patients undergoing HSCT, with a 12% to 42% mortality rate (Averbuch et al., 2013). There are studies and guidelines that mention the importance of timely and effective selection of an appropriate antibiotic regimen (Chinese Society of Hematology, C. M. A. and Chinese Medical Doctor Association, 2020; Daniels et al., 2019). Since the clinical manifestations of infection in hematology patients are often atypical and the infections are usually severe, timely and accurate use of effective antimicrobial regimens is important, which relies on the rapid identification of the causative microorganism (Valdez et al., 2011).

However, conventional microbiological tests (CMTs), such as culture, serology polymerase chain reaction (PCR) and immunology tests, often have difficulty guiding the timely selection of appropriate antimicrobial regimens due to their time consumption, low positivity rates, and narrow pathogen coverage (Gu et al., 2021). Although the positive rate of blood culture in patients with febrile neutropenia is higher than that in patients without febrile neutropenia, it is only 10-25%, and blood culture cannot detect viruses and has a low rate of fungal detection (Gustinetti and Mikulska, 2016). Therefore, a new approach that can rapidly and accurately detect a wide range of pathogens is urgently needed.

In recent years, metagenomic next-generation sequencing (mNGS) has rapidly emerged in the field of pathogenic microorganism detection (Zhong and Yang, 2023), which covers a wide range of pathogens, such as viruses, bacteria, fungi, and parasites, as long as the sample contains detectable DNA or RNA. mNGS has significant advantages over CMT, such as higher positive detection rates, identification of mixed infections, and the ability to detect atypical pathogens (Han et al., 2019). A meta-analysis illustrated that the diagnostic efficacy of mNGS varied depending on the sample, with a sensitivity and specificity of 90% and 86% for blood, 75% and 96% for cerebrospinal fluid, and 84% and 67% for orthopedic samples, respectively (Govender et al., 2021).

Several studies have shown that mNGS has advantages in hematology patients with infection (Liu W. D. et al., 2021; Liu W. et al., 2021; Fu et al., 2022), which has high positivity rate, sensitivity and specificity of pathogen detection. And there have been studies showing that mNGS has superiority in the detection of viruses, fungi, and especially atypical pathogens (Huang et al., 2020). In a retrospective study by C. Xu et al (Xu et al., 2022), mNGS with plasma was advantageous over CMT in hematology patients with a higher positive detection rate (72.6% vs. 31.4%, P < 0.001) and had a greater positive impact in the neutropenia group in terms of both diagnosis and treatment (diagnosis: 54.3% vs. 40.5%, P = 0.013; treatment: 45.7% vs. 30.7%, P = 0.004). There have also been some studies on the clinical use of mNGS with various samples in patients with hematopoietic stem cell transplantation (HSCT) (Sun J. H. et al., 2022; Zhang X. et al., 2022; Shen et al., 2023). However, there are few comprehensive studies on the clinical value of mNGS with plasma in different populations of hematology patients, such as those with or without neutropenia and those with or without HSCT. Therefore, this study focused on the clinical value of mNGS using plasma in different populations of hematological patients.

## 2 Materials and methods

### 2.1 Study design

We retrospectively collected information on patients who were hospitalized in the Department of Hematology at West China Hospital of Sichuan University and underwent testing for mNGS with plasma from October 2020 to July 2023. The inclusion criteria were as follows: (1) hematologic diseases; (2) suspicion of infection based on clinical symptoms and first mNGS test with plasma during one hospitalization; and (3) other relevant conventional microbiological tests, such as culture, (1-3)-β-D-glucan test (G test), GM test, and polymerase chain reaction (PCR) was completed within 3 days and 7 days of the mNGS test. The exclusion criteria were as follows: (1) repeat test after the first mNGS test during one hospitalization; (2) hematological diseases were ruled out; (3) incomplete patient information or medical records; and (4) lack of paired conventional microbiological tests (CMTs). Neutropenia was defined as an absolute neutrophil count <500/μL or <1000/μL with a high probability of decrease <500/μL in the next 2 days. In addition, we also collected patients’ clinical characteristics, clinical signs and symptoms, laboratory tests, imaging tests, treatment procedures and prognosis. This retrospective study was approved by the West China Hospital of Sichuan University Ethics Committee (2023-890).

### 2.2 CMT

The CMT performed by the patient was chosen by the clinician based on the patient’s clinical symptoms, signs, and imaging studies. We collected relevant CMT based on the patient’s clinical signs and symptoms according to IDSA (Freifeld et al., 2011), including cultures, smear, (1-3)-β-D-glucan test (G test), GM test, antigen detection, enzyme-linked immunospot assay (T-SPOT), Epstein–Barr virus (EBV) and cytomegalovirus (CMV) PCR detection within 3 and 7 days of mNGS specimen collection. Pharyngeal swabs are only used for the detection of respiratory viruses and atypical pathogens (including influenza A/B viruses, adenoviruses, respiratory syncytial viruses, rhinoviruses, COVID-19, chlamydia and mycoplasma) by PCR. PCR detection of EBV and CMV in cases of viremia. G test and GM test and fungal culture in case of fungal infection. CMT was performed according to clinician selection and standard procedures.

### 2.3 Metagenomic next-generation sequencing detection

The patients’ plasma samples were collected and sent to Precision Medicine Key Laboratory of Sichuan Province and Precision Medicine Center for testing. A TIAN amp Micro DNA Kit (DP316, TIANGENBIOTECH, Beijing, China) was used to extract DNA. The Agilent2100 Bioanalyzer was used for quality control libraries with fragment sizes of 200-300 bp. QubitdsDNAHS Assay Kit (Thermo Fisher Scientific Inc.) to control the concentration of the DNA libraries. High-throughput mNGS sequencing was performed using MGI2000. Raw results from sequencing were preferred to remove low-quality and splice contamination data, and the filtered data were compared to the BWA (http://biobwa.sourceforge.net/) to remove human reference genome sequences (Ruan et al., 2022). The remaining data were removed from low-complexity sequences and then compared to the BGI Microbial Reference Database PMDB (including 6350 bacterial, 1064 fungal, 4945 viral, 234 parasitic species of fungi, 4945 species of viruses, 234 species of parasites) (Miao et al., 2018). Combining the detection of negative control samples to exclude contamination and implausible results, the generic exclusion criteria for all species are as follows: (1) RPM of the species < 3 times the RPM of the species in the negative control samples; (2) common known contaminant species; (3) number of sequences < 3 at the species level; (4) uneven distribution of the species compared to the reference genome, as the distribution is too concentrated and may only be a random fragment of a region that are not true sequences; (5) common definitive bacteria in the respiratory tract reported in the literature; (6) strong positive detections of the species exist in other samples in the same batch (sequence number > 10,000, or sequence number much greater than all other samples), combined with sample extraction order to exclude. (7) Common environmental species that are not pathogenic. After excluding contamination and implausible results, due to the large number of species detected by bacteria, then ranked according to the sequence number at the genus level, only the top 10 genera were considered for general species, and only the top 2 species in each genus were considered for sequence number (Miao et al., 2018); for common species that are very pathogenic (e.g., *Klebsiella pneumoniae*), they were not subject to this ranking common species that are highly pathogenic (e.g., *Klebsiella pneumoniae*) are not subject to this ranking and are retained as long as they meet the above exclusion criteria; fungi and pathogens, as long as they meet the above exclusion criteria, are considered. viruses are retained as long as they meet the above exclusion criteria; parasites are retained because of their parasites due to larger genome sequences and similarity to the human reference genome and higher similarity to the human reference genome. In addition to the above exclusion criteria, they also need to meet the sequence number > 10 (Miao et al., 2018).

### 2.4 Diagnosis of infection

Two experienced clinicians independently and retrospectively evaluated the patient’s clinical symptoms, laboratory tests, radiological manifestations, CMT, and treatment response. And the diagnosis of different types of infection was retrospectively made with reference to the CDC/NHSN Surveillance Definitions for Specific Types of Infections.

### 2.5 Clinical relevance of mNGS results

Considering that there is no uniform standard for the interpretation of the results of mNGS, two experienced hematology clinicians individually and retrospectively evaluated the patient’s clinical symptoms, laboratory tests, radiological manifestations, CMT, and treatment response, and categorized the positive results of mNGS as follows: (1) definite: compatible clinical features; consistent with CMT results; (2) probable: compatible clinical features; inconsistent with CMT results; initial antibiotic regimen did not cover pathogenic microorganisms; antibiotic regimen adjusted by mNGS and be effective within 7 days; (3) possible: compatible clinical features; inconsistent with CMT results; initial antibiotic regimen covered pathogenic microorganisms; antibiotic regimen adjusted by mNGS including adjustment of drug dosage and frequency, suspicion of antibiotic resistance, discontinuation of unrelated antibiotics and be effective within 7 days; (4) likely: compatible clinical features; inconsistent with CMT results; initial antibiotic regimen covered pathogenic microorganisms; continuation of initial antibiotic regimen and be effective within 7 days; (5) unlikely: incompatible clinical features or non-pathogenic microorganisms or antibiotics had covered but treatment was ineffective. If the mNGS result was negative, the clinician synthesized to determine the clinical significance of the result. Definition of antibiotic adjustment: initialization of the appropriate antibiotics, reduction of the use of unnecessary antipathogenic microbial drugs, addition of new types of antipathogenic microbial drugs, replacement of antibiotics with new antibiotics or dosage and frequency adjustment of antipathogenic microbial drugs within three days of receiving the mNGS results.

### 2.6 Assessment of efficacy

The efficacy of the antimicrobial regimens was defined as follows: (1) clinical symptoms: patients’ peak temperature decreasing by 0.5°C, or there being no fever after the adjustment of antipathogenic microorganism regimes, as well as improvement of primary manifestations at the site of the associated infection, such as cough, sputum, abdominal pain, diarrhea, urinary frequency, urinary urgency, etc.; (2) laboratory tests: decrease in white blood cell counts and neutrophil percentages, and decrease in markers of infection, including procalcitonin (PCT), C-reaction protein (CRP), interleukin-6 (IL-6); (3) imaging improvement: chest X-ray or CT scan. Two experienced clinicians independently determined the effectiveness of the antimicrobial regimens.

### 2.7 Statistical analysis

Categorical variables were reported using frequencies and percentages. Continuous variables that are normally distributed are presented using the mean ± standard deviation (SD); otherwise, they are presented using the median ± quartile spacing. Contingency tables (2×2) were established to calculate the sensitivity, specificity, positive predictive value (PPV), negative predictive value (NPV), positive likelihood ratio (PLR), and negative likelihood ratio (NLR). Data analyses were performed using SPSS 26.0 software. Figures were rendered using GraphPad Software (version 8.02). Comparative analysis was conducted by the t test, Pearson χ2 test, McNemar test, or Fisher exact test. P values <0.05 were considered significant.

## 3 Results

### 3.1 Basic characteristics

**Figure 1:**
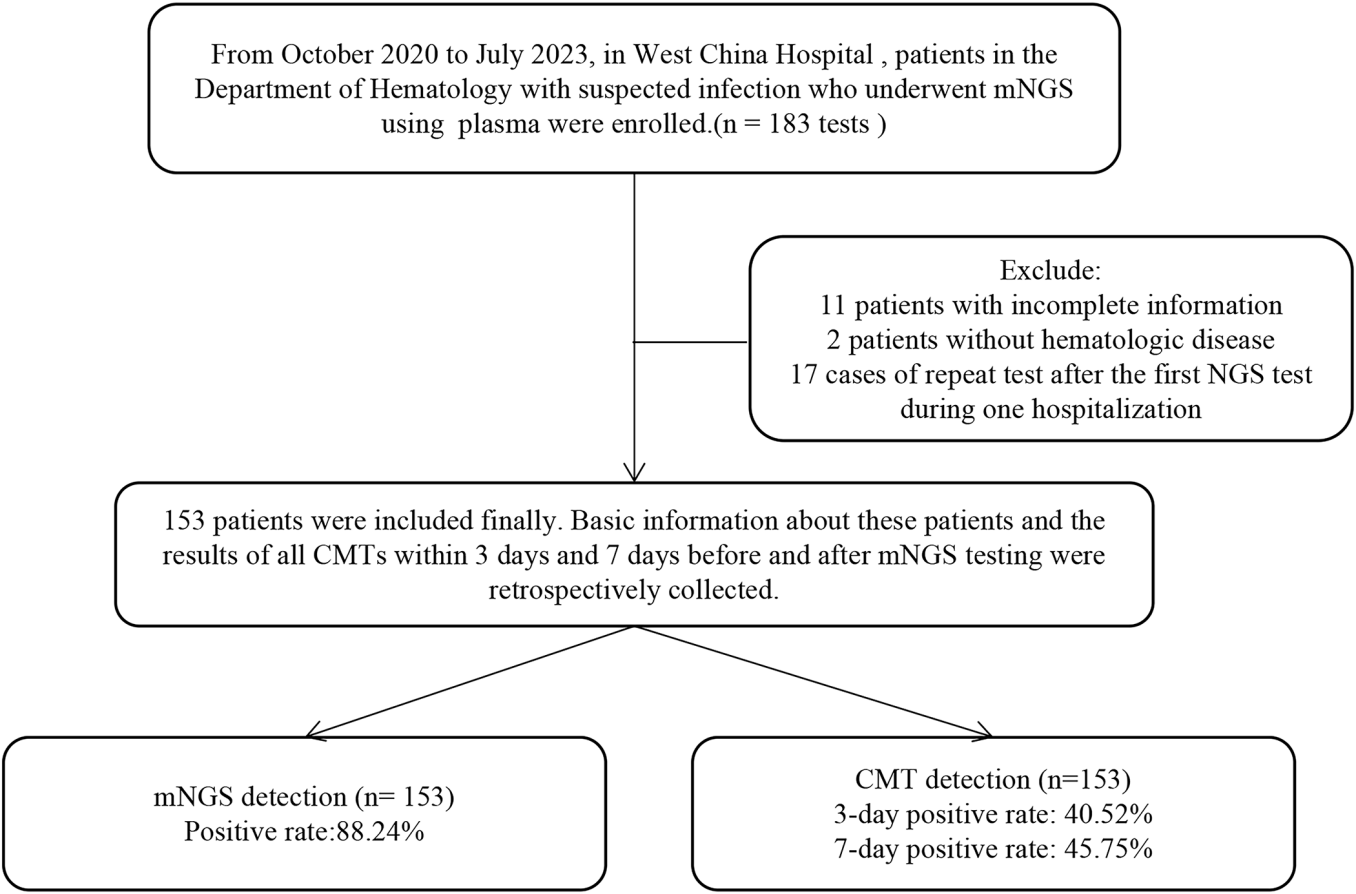
Flow diagram of the patients included in the study. CMT, conventional microbiological test.

From October 2020 to July 2023, we screened all patients who underwent mNGS with plasma at the Department of Hematology, West China Hospital. Based on the exclusion and inclusion criteria, 153 patients were ultimately enrolled in this retrospective study (**Figure 1**). For patients with mNGS performed multiple times during a single hospitalization, we included cases in which the first mNGS was performed. We enrolled 153 hematology patients, including 84 males (54.90%) and 79 females (51.63%), with a median age of 40, ranging from 15 to 84 years old. The underlying diseases included 94 (32.03%) AML, 29 (18.95%) ALL, 8 (5.23%) MDS, 5 (3.27%) AA, 12 (7.84%) MM, 33 (21.57%) NHL, 3 (1.96%) NHL, and 14 (9.15%) other diseases. Thirteen (8.50%) patients had concomitant hemophagocytic lymphohistiocytosis. Of these patients, 141 (92.16%) had been treated with chemotherapy, 71 (46.41%) with molecularly targeted drugs, 33 (21.57%) with anti-drug antibodies, and 52 (33.99%) with hematopoietic stem cell transplant (HSCT). At the time of the mNGS test, 47.06% (72/153) of the patients had neutropenia. A total of 208 infections occurred, including 86 pulmonary infections, 79 bloodstream infections, 15 abdominal infections, 8 gastrointestinal tract infections, 8 oral and perianal mucosa infections, 2 urinary tract infections, 6 soft tissue infections, 2 protozoan infections and 2 hepatitis B virus infections. The majority of patients (150/153, 98.04%) were exposed to antibiotics prior to sampling. The 28-day mortality rate was 29.41% (45/153). The detailed demographic characteristics, underlying disease and infection distribution, laboratory examination results and other information are summarized in **Table 1**.

**Table 1:** Demographic and clinical characteristics in this study.

Patients, n (%)	153(100.00%)
Male	84(54.90%)
Female	79(51.63%)
Age(y)	
median (range)	49(15-84)
Diagnosis, n (%)	
AML	49(32.03%)
ALL	29(18.95%)
MDS	8(5.23%)
AA	5(3.27%)
MM	12(7.84%)
NHL	33(21.57%)
HL	3(1.96%)
Other disease	14(9.15%)
With HLH, n (%)	
Yes	13(8.50%)
No	140(91.50%)
Therapy of underlying diseases, n (%)	
Chemotherapy	141(92.16%)
Molecular targeted drugs	71(46.41%)
Anti-Drug antibody	33(21.57%)
HSCT	52(33.99%)
Neutropenia	
Yes	72(47.06%)
No	81(52.94%)
Days of hospitalization	
median (range)	45(1-179)
Laboratory examination, median (range)	
PLT	29(1-513)
Hb	76(48-127)
WBC, 10^9/L	1.28(0-91.11)
ANC, 10^9/L	0.36(0-51.99)
CRP, mg/L	76(0-604)
PCT, ng/mL	0.49(0.03-98.3)
IL-6	79.9(1.5-5000)
Infections, n (%)	
Pulmonary infection	86(41.35%)
BSIs	79(37.98%)
Abdominal infection	15(7.21%)
The gastrointestinal tract infection	8(3.85%)
Oral and perianal mucosa infection	8(3.85%)
Urinary tract infection	2(0.96%)
Soft tissue infection	6(2.88%)
Protozoan infection	2(0.96%)
hepatitis B virus infection	2(0.96%)
Uncertain	6
Non-infection	3
Previous antibiotic exposure, n (%)	
Yes	150(98.04%)
No	3(1.96%)
28-day mortality, n (%)	45(29.41%)

AML, Acute myeloid leukemia; ALL, Acute lymphocytic leukemia; MDS, Myelodysplastic syndrome; MM, Multiple myeloma; NHL, Non-Hodgkin’s lymphoma; HSCT, Hematopoietic stem cell transplantation; HLH, Hemophagocytic Lymphohistiocytosis; BSIs, Bloodstream infections.

### 3.2 Distribution of pathogens detected by mNGS and CMT

**Figure 2:**
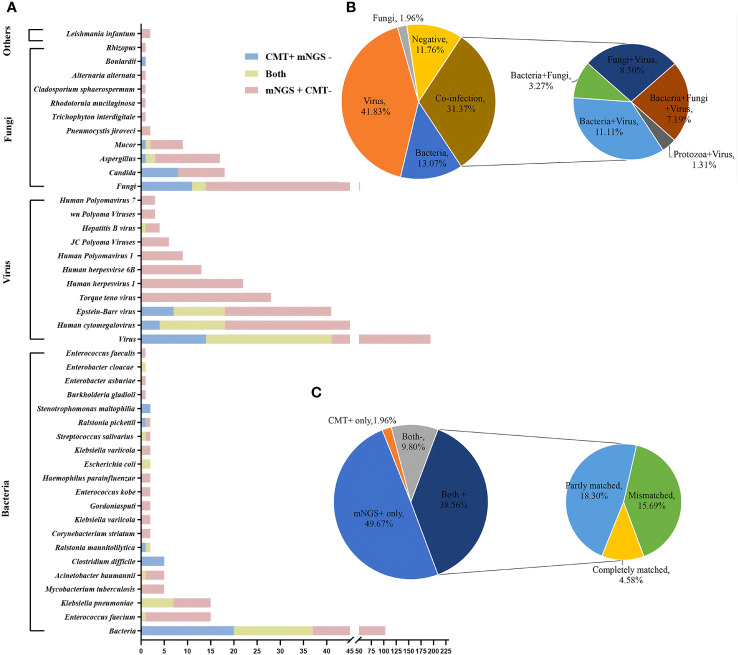
Distribution of pathogens identified in hematology patients using CMT versus mNGS. **(A)** The figure shows the number of pathogenic microorganisms detected by mNGS and CMT. The blue bar indicates positivity for CMT and negativity for mNGS, the yellow bar indicates positivity for both, and the pink bar indicates positivity for mNGS and negativity for CMT. **(B)** Distribution of microorganism types detected by mNGS using plasma. **(C)** Concordance between mNGS and CMT.

Pathogenic species detected by mNGS in 153 plasmas consisted of 39 bacteria, 19 viruses, 19 fungi and 2 parasites, while the CMT detected 24 bacteria, 6 viruses, 8 fungi and 1 parasite. According to the NGS results, viruses (64/153, 41.8%) were the most common pathogens identified, followed by bacteria (20/153, 13.1%). The top 10 most frequent bacterial pathogens detected by mNGS included *Enterococcus faecium* (15/153), *Klebsiella pneumoniae* (15/153), *Mycobacterium tuberculosis* (5/153), *Acinetobacter baumannii* (5/153), *Corynebacterium striatum* (2/153), *Klebsiella variicola* (2/153), *Gordoniasputi* (2/153), *Enterococcus kobe* (2/153), *Haemophilus parainfluenzae* (2/153), and *Escherichia coli* (2/153), and all were clinically relevant. The top five viruses detected by mNGS were *Human cytomegalovirus* (42/153), *Epstein-Barr virus* (34/153), *Torque teno virus* (28/153), *Human herpesvirus1* (22/153), *Human herpesvirseand human herpesvirus 6B* (13/153), and the top four fungi were *Aspergillus* (16/153), *Candida* (10/153), *Mucor* (8/153), and *Pneumocystis jiroveci* (2/153). The pathogen spectrum detected by CMT revealed that *Klebsiella pneumoniae* (7/153), *Clostridium difficile* (5/153), *Ralstonia mannitolilytica* (2/153), *Escherichia coli* (2/153), and *Stenotrophomonas maltophilia* (2/153) were the leading bacterial pathogens, and *Epstein-Barr virus* (18/153), *Human cytomegalovirus* (18/153) were the dominant viral pathogens. CMT detected 8 cases *Candida*, 3 *Aspergillus* and 2 *Mucor*. It is worth mentioning that there were 11 positive G tests (11/153) and 7 positive GM tests (7/153) within 7 days of the mNGS test. More information on the pathogen distribution detected by mNGS and CMT is shown in **Figure 2A**.

Of these 153 specimens, mNGS detected one type of microorganism (either bacteria, fungi or virus) in 87 cases (56.86%), two types of microorganisms in 37 cases (24.18%), three types of microorganisms in 11 patients (7.19%) and negatives in 18 cases (11.76%). According to mNGS, bacteria and virus “co-infection”, bacteria and fungi “co-infection”, fungi and virus “co-infection” and protozoa and virus “co-infection” accounted for 11.11% (n = 17), 3.27% (n = 5), 8.50% (n = 13) and 1.31% (n = 2), respectively. Bacteria and fungi and virus “co-infection” accounted for 7.19% (n = 11) (**Figure 2B**).

In our 153 cases, 76 cases (76/153, 49.67%) were positive for mNGS and negative for CMT, and 3 cases (3/153, 1.96%) were positive for CMT and negative for mNGS. The results of mNGS and CMT were both positive in 59 cases (59/153, 38.56%), of which 7/59 (11.86%) were completely matched, 28/59 (47.46%) were partly matched, and 24/59 (40.68%) were mismatched. Both were negative in 9.80% (15/153) of cases (**Figure 2C**).

### 3.3 Comparison of mNGS and CMT detection positivity rates

**Figure 3:**
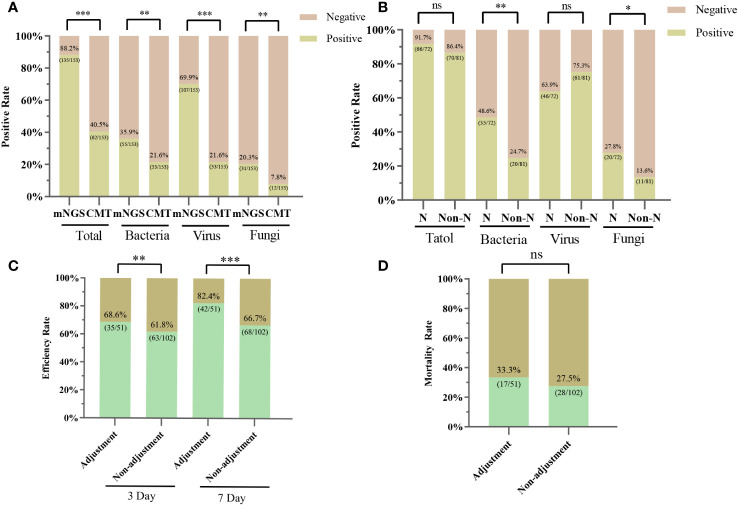
Positive rates for mNGS and CMT pathogens and the clinical impact of mNGS. **(A)** Positive rates of mNGS and CMT for overall, bacterial, viral, and fungal detection, respectively. **(B)** Positive rates of mNGS for bacterial, viral and fungal detection in the neutropenia and non-neutropenia groups. N, neutropenia group; Non-N, non-neutropenia group. **(C)** 3-day and 7-day efficiency rates for the adjustment and non-adjustment groups. **(D)** 28-Day mortality rate for the adjustment and non-adjustment groups; ∗ P < 0.05; ∗∗ P < 0.01; ∗∗∗ P < 0.001; ns, not statistically significant.

Among the 153 plasma samples, the positive rate of mNGS was 88.24% (135/153), which was significantly higher than that of CMT (40.52%, 62/153) (P<0.001). We also counted the positivity rates of different pathogen types. For bacteria, the positive rates for mNGS and CMT were 35.95% (55/153) and 21.57% (33/153), respectively (P < 0.005). The positive rate of mNGS for virus detection was 69.93% (107/153), which was significantly higher than that of CMT (21.57%, 33/153) (P<0.001). For the detection of fungi, the positive rate of mNGS was slightly higher than that of CMT (20.26%, 31/153 vs. 7.84%, 12/153) (P < 0.01) (**Figure 3A**). Hematology patients often have neutropenia, and we also counted the positivity rate of mNGS for various pathogens in the neutropenia and non-neutropenia groups separately. The positive rate of detection of bacteria and fungi was higher in the neutropenia group than in the non-neutropenia group (47.95% vs. 24.69%, P<0.01; 27.78% vs. 13.58%, P<0.05). For the detection of the virus, the positive rates of the two groups were similar and not statistically significant (63.9% vs. 75.3%, P=0.124) (**Figure 3B**). For the HSCT and non-HSCT groups, the positivity rates for the various pathogens were similar, and the difference was not statistically significant. See **Supplementary Table S1** in the electronic **Supplementary Material** for details.

### 3.4 Diagnostic performance of mNGS and conventional microbiological tests

We calculated the diagnostic value of mNGS when CMT or blood culture or EBV-PCR or CMV-PCR was the gold standard. The comparison of the sensitivity, specificity, PLR, NLR, PPV, and NPV of mNGS is shown in **Table 2**. When CMT was used as the reference standard, the sensitivity and specificity of mNGS were 62.5% (95% CI: 48.5%-74.8%) and 17.53% (95% CI: 10.8%-26.9%), respectively. When we used blood culture, CMV-PCR, and EBV-PCR as reference standards, the sensitivity of mNGS was 83.3% (95% CI: 57.7%-95.6%), 61.9% (95% CI: 38.7%-81.0%), and 70.0% (95% CI: 45.7%-87.2%), and the specificity of mNGS was 71.5% (95% CI: 62.3%-79.3%), 89.1% (95% CI: 77.1%-95.5%), and 80.4% (95% CI: 67.2%-89.3%), respectively.

**Table 2:** Diagnostic value of mNGS.

Test system/Statistics	CMT		Blood Culture		EBV-PCR		CMV-PCR	
	(+)	(-)	(+)	(-)	(+)	(-)	(+)	(-)
mNGS (+)	35	80	15	33	13	6	14	11
mNGS (-)	21	17	3	83	8	49	6	45
Sen (95% CI)	62.5% (48.5%-74.8%)		83.3% (57.7%-95.6%)		61.9% (38.7%-81.0%)		70.0% (45.7%-87.2%)	
Spe (95% CI)	17.5% (10.8%-26.9%)		71.5% (62.3%-79.3%)		89.1% (77.1%-95.5%)		80.4% (67.2%-89.3%)	
PLR (95% CI)	0.76 (0.61-0.95)		2.93 (2.05-4.18)		5.67 (2.48-12.97)		3.56 (1.95-6.51)	
NLR (95% CI)	2.14 (1.39-3.30)		0.23 (0.08-0.66)		0.43 (0.25-0.74)		0.37 (0.19-0.74)	
PPV (95% CI)	30.4% (22.4%-39.8%)		31.3% (19.1%-46.4%)		68.4% (43.5%-86.4%)		56.0% (35.3%-75.0%)	
NPV (95% CI)	44.7% (29%-61.5%)		96.5% (89.4%-99.1%)		86.0% (73.7%-93.3%)		88.2% (75.4%-95.1%)	

mNGS, metagenomic next-generation sequencing; CMT, conventional microbiological test; CI, confidence interval; Sen, sensitivity; Spe, specificity; PLR, positive likelihood ratio; NLR, negative likelihood; PPV, positive predictive value; NPV, negative predictive value.

### 3.5 Evaluation of clinical treatment and prognosis of mNGS

**Figure 4:**
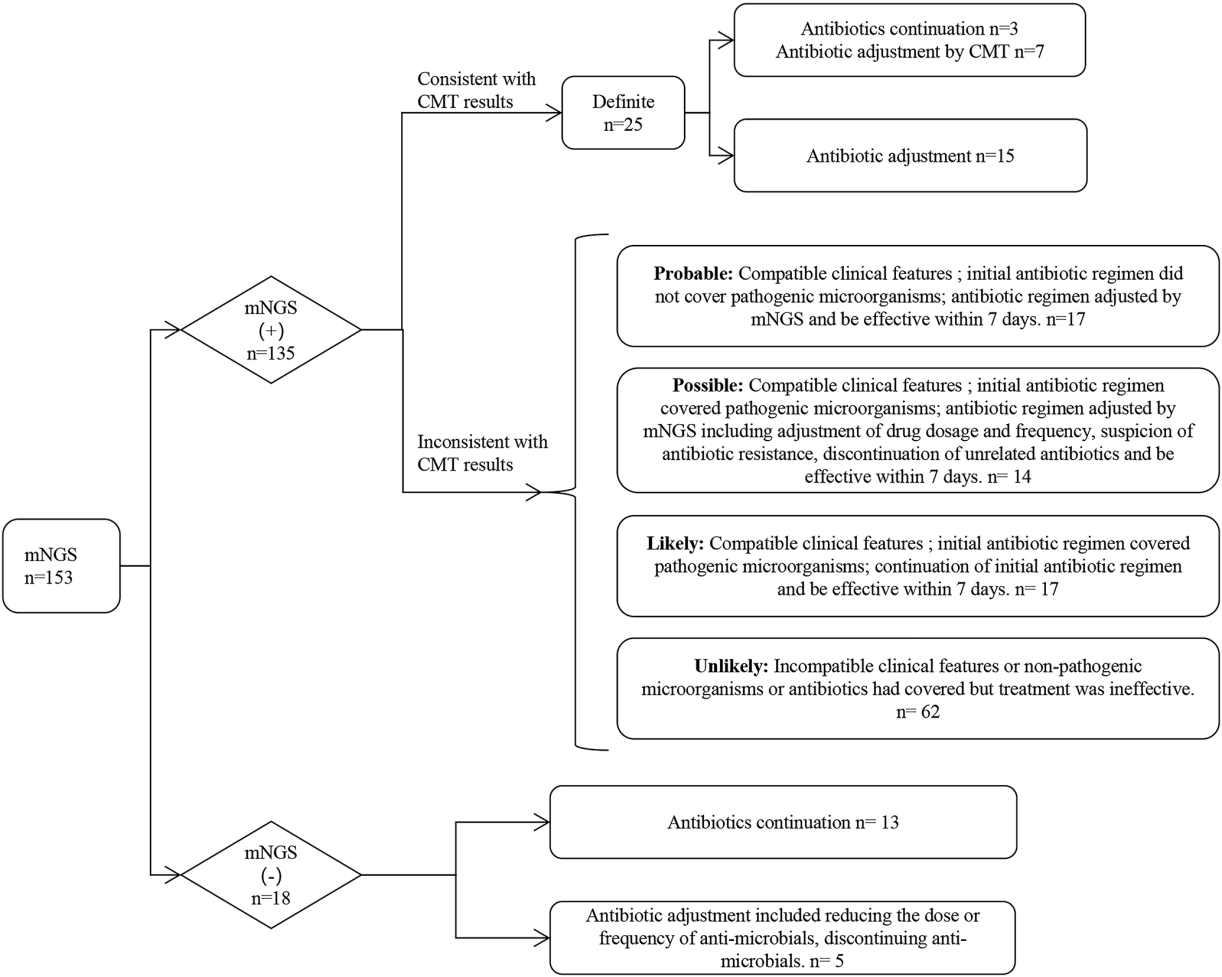
Flowchart of patients’ antimicrobial regimens based on mNGS reports.

Whether the results of mNGS were credible or not, we determined the compatibility of CMT, pathogenicity, clinical characteristics, and response to treatment (**Figure 4**). Of these 153 patients, 16.34% (25/153) had results consistent with the CMT and were determined to be “definite”, with 22 antibiotic continuations and 3 antibiotic adjustments. The fraction of non-compliance with CMT was composed of “Probable”, “Possible”, “Likely” and “Unlikely” in 17 cases (11.11%), 14 cases (9.15%), 17 cases (11.11%), and 62 cases (40.52%), respectively. mNGS was negative in 18 cases (11.76%), of which antibiotics were continued in 13 cases (8.50%) and antibiotic adjustments were made in 5 cases (3.27%). Overall, 51 (33.33%) were antibiotic adjusted by mNGS, and 102 (66.67%) were not adjusted. Differences between the adjustment and non-adjustment groups are shown in **Supplementary Table S2**. The effectiveness of anti-infective therapy was determined by whether the patient’s clinical manifestations improved and whether inflammatory markers (CRP, PCT, IL-6) decreased. The 3-day efficiency rate in the adjustment group was similar to that in the non-adjustment group (68.63% vs. 61.76%, P = 0.40), but the 7-day efficiency rate was higher than that in the non-adjustment group (82.35% vs. 66.67%, P < 0.05) (**Figure 3C**). However, the 28-day mortality rate was similar in both groups (33.33% vs. 27.45, P > 0.05) (**Figure 3D**).

### 3.6 Clinical applications of mNGS in HSCT and non-HSCT, neutropenia and non-neutropenia patients

**Figure 5:**
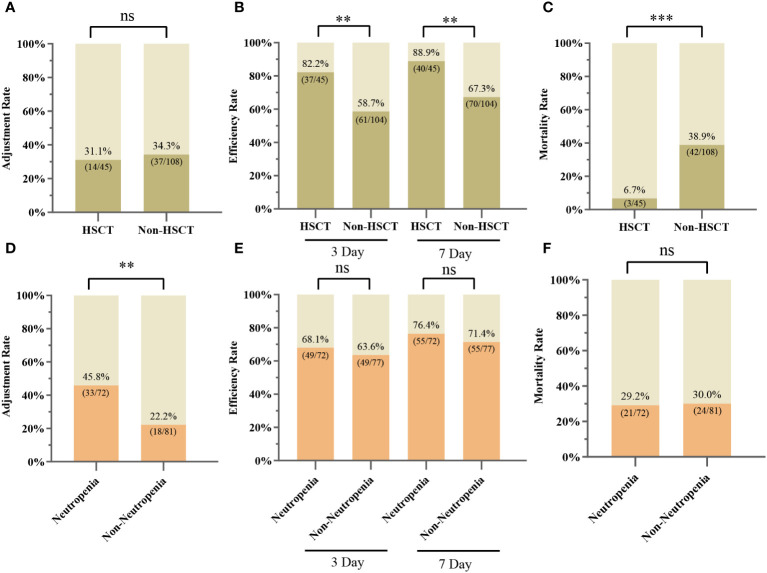
Clinical applications of mNGS in different subgroups. **(A)** Adjustment rate for the HSCT and non-HSCT groups. **(B)** 3-day and 7-day efficiency rates for the HSCT and non-HSCT groups. **(C)** 28-Day mortality rate for the HSCT and non-HSCT groups. **(D)** Adjustment rate for neutropenia and non-neutropenia groups. **(E)** 3-day and 7-day efficiency rates for the neutropenia and non-neutropenia groups. **(F)** 28-Day mortality rate for neutropenia and non-neutropenia groups; ∗∗ P < 0.01; ∗∗∗ P < 0.001; ns, not statistically significant.

Of our 153 patients, 45/153 (29.41%) and 108/153 (70.59%) patients were HSCT and non-HSCT patients, respectively, with similar adjustment rates in both groups (31.11% vs. 34.26%, P > 0.05) (**Figure 5A**). Differences between the HSCT and non-HSCT groups are shown in **Supplementary Table S3**. The 3-day and 7-day efficacy rates were higher in the HSCT group than in the non-HSCT group (3-day: 82.22% vs. 58.65%, P < 0.01; 7-day: 88.89% vs. 67.31%, P < 0.01) (**Figure 5B**). Moreover, 28-day mortality was significantly lower in the HSCT group than in the non-HSCT group (6.67% vs. 38.89%, P < 0.000) (**Figure 5C**). Patients were categorized into a neutropenia group (72/153) and non-neutropenia group (81/153). The antibiotic adjustment rate was higher in the neutropenia group than in the non-neutropenia group (45.83% vs. 22.22%, P < 0.01) (**Figure 5D**). The 3-day and 7-day efficacy rates between the two groups were slightly higher in the neutropenia group than in the non-neutropenia group (3-day: 68.06% vs. 63.64%; 7-day: 76.39% vs. 71.43%), although the difference was not statistically significant (P > 0.05) (**Figure 5E**). The mortality rates at 28 days were similar in the two groups (29.17% vs. 29.63%, P > 0.05) (**Figure 5F**).

## 4 Discussion

Timely, rapid, and accurate identification of pathogens is critical to the treatment and prognosis of hematology patients on antimicrobial regimens. mNGS is a rapid, efficient, and unbiased technique for obtaining pathogen nucleic acid sequence information and has recently been widely used in clinical practice to assist in the detection of pathogens (Simner et al., 2018). This retrospective study comprehensively evaluated the diagnostic value of mNGS with plasma for infection in different subgroups of hematology patients and assessed the impact of mNGS on treatment and prognosis.

In this study, the distribution of pathogens detected by mNGS and CMT was consistent with previously published epidemiology of hematological pathogens (Cai et al., 2023). The most common bacteria include *Enterococcus faecium*, *Klebsiella pneumoniae*, *Mycobacterium tuberculosis* and *Acinetobacter baumannii*, which are all common microorganisms in hematology patients (Ren et al., 2021). Early detection of these pathogens helps in diagnosis and treatment. However, for viral testing, CMV, EBV and *Torque teno virus* ranked as the top three viruses detected, and this is in general agreement with the spectrum of viral infections reported by P. Zhang et al. (Zhang P. et al., 2022). It has also been tested for common pathogenic fungi in hematology patients, such as *Aspergillus*, *Mucor, and Pneumocystis japonicum*, which are difficult to culture in blood cultures. It is worth mentioning that 5 cases of *Mycobacterium tuberculosis* infection and 2 cases of *Leishmania infantum* infection were detected in this study, illustrating the effective diagnostic value of mNGS for the detection of atypical pathogens, which are more common in hematology patients than in the general population (Zhang et al., 2023). Simple viral and bacterial detection rates were significantly higher than simple fungal detection rates (41.83% vs. 13.07% vs. 1.96%), which compensated for disadvantages of CMT in virus detection. Viral load has been previously reported to be relevant for the severity of illness and survival in patients with sepsis (Duan et al., 2021), which highlights the benefit of mNGS. It has been reported that immunosuppressed patients are susceptible to mixed infections (Zhang B. et al., 2022). Our study showed that 38.56% of plasma had mixed infections, which indicated that mNGS with plasma had a significant advantage in the diagnosis of polymicrobial infection (Dropulic and Lederman, 2016).

The results showed that the overall positive detection rate of mNGS was significantly higher than that of CMT (88.24% vs. 40.52%, P<0.001). Moreover, mNGS was superior to CMT in detecting fungi (20.26% vs. 7.84%, P<0.01), especially in patients with neutropenia (27.78% vs. 13.58%, P<0.05), which is a common infection in hematology patients due to immunosuppression, the use of cytotoxic drugs and suppression of the bone marrow by the primary disease (Lionakis et al., 2014). Patients with neutropenia are more susceptible to fungal infections due to their weakened immune function (Pagano et al., 2009). A previous study (Yang et al., 2021) showed the high diagnostic value of mNGS using lung biopsy (sensitivity: 80.0%) or bronchoalveolar lavage fluid (sensitivity: 84.4%) to diagnose pulmonary fungal infection. However, because hematology patients often do not tolerate invasive procedures, plasma can be used as a complementary test to diagnose fungal infections. Zhang et al. (Zhang M. et al., 2022) reported that mNGS had more diagnostic advantages in patients with febrile neutropenia (FN). In our study, the positive rate of detection of bacteria was higher in the FN group than in the non-FN group (47.95% vs. 24.69%, P<0.01), which was consistent with previously reported results (Hao et al., 2022). This study illustrated that mNGS with plasma played a more important role in patients with neutropenia.

It is worth mentioning that 98.04% of patients received antibiotics at the time of mNGS in this study, which usually affected the detection positive rate of CMT. Previous studies have reported that the positivity rate of mNGS was not affected by whether or not they had undergone antibiotic exposure (Zhang M. et al., 2022), and our results were similar to a study reported by P. Parize et al. (Parize et al., 2017), which was a prospective study in which patients were not exposed to antibiotics.

When CMT was used as the reference standard, the sensitivity and specificity were 62.5% and 17.53%, respectively, which were inferior to the results reported by S. F. Hao et al. (Hao et al., 2022) (sensitivity: 75.68%; specificity: 36.07%) and the results reported by C. Xu et al. (Xu et al., 2022) (sensitivity: 79.6%; specificity: 36.8%). The major reason might be that a large proportion of mNGS-positive specimens were negative for CMT in our study, and we included CMT results within 3 days of mNGS, which would further reduce the positivity rate of CMT. We further analyzed the reason for this is related to the high detection rate of viruses, whereas there are some limitations in traditional laboratory tests for viral detection (Gu et al., 2019), and therefore, there is a lack of validated CMT as a reference for the viral positivity of the specimens detected by mNGS with plasma. When blood culture was used as the reference standard, the sensitivity and specificity of mNGS were 83.3% and 71.5%, respectively, which demonstrated that mNGS has good diagnostic value for bloodstream infections. Considering that virus clearance was difficult in a short period of time, when we used EBV-PCR and CMV-PCR within 7 days of mNGS as the reference standard, the sensitivity was 61.9% and 70.0%, and the specificity was 89.1% (77.1%-95.5%) and 80.4% (67.2%-89.3%), respectively. Compared to CMT, mNGS was able to detect EBV and CMV more accurately and sensitively, even when conventional PCR failed to detect them. Overall, mNGS with plasma showed good diagnostic value for viral and bacterial bloodstream infections.

To better assess the relevance of mNGS results to clinical practice, we performed a standardized evaluation process. Of these 135 mNGS-positive patients, 73/135 (54.07%) cases were ultimately considered clinically compatible, with 62/135 (45.92%) cases considered nonpathogenic or not clinically relevant. Patients were categorized into an adjustment group non-adjustment group based on whether or not antibiotics were adjusted by mNGS. The baseline characteristics of the two groups have been added to the **Supplementary Material**. The 3-day efficacy rates were not significantly different between the two groups (68.6% vs. 61.8%, P = 0.40), but the 7-day efficacy rates were higher in the adjusted group than in the non-adjusted group (82.35% vs. 66.67%, P < 0.05), suggesting that adjusting antibiotics by mNGS would benefit patients in the long term. Although patients in the adjustment group were sicker than those in the non-adjustment group, the final mortality rate was similar in both groups.

mNGS demonstrated a greater advantage in the group of patients with hematopoietic stem cell transplantation (HSCT). With the complexity of infection factors after HSCT (Sahu, 2021) and more aggressive clinical management of infections. In our study, although the adjustment rates were similar between the HSCT and non-HSCT groups (31.11% vs. 34.26%, P > 0.05), both the 3-day and 7-day efficacy rates in the HSCT group were higher than those in the non-HSCT group (3-day: 82.22% vs. 58.65%, P < 0.01; 7-day: 88.89% vs. 67.31%, P < 0.01), and the 28-day mortality rate was lower in the HSCT group than in the non-HSCT group (6.67% vs. 38.89%, P < 0.000). This study demonstrates the advantages of mNGS in HSCT patients, allowing timely adjustment of antimicrobial regimens, improving patient prognosis and reducing mortality rates.

Hematology patients often have neutropenia due to primary disease, high doses of radiotherapy, transplantation, and the use of new small molecule drugs (Freifeld et al., 2011). Patients with neutropenia are more susceptible to invasion by pathogens, and in our study, patients with neutropenia had a higher rate of testing positive for microorganisms, which is consistent with previously reported results (Guo et al., 2022). Infection progresses more rapidly in patients with neutropenia (Sun Y.-Q. et al., 2022), and clinicians are more likely to be aggressive and stringent in anti-infective therapy for patients with neutropenia. In our study, the antibiotic adjustment rate was higher in the neutropenia group than in the non-neutropenia group (45.83% vs. 22.22%, P < 0.01), and after adjustments to the treatment regimen, the neutropenia and non- neutropenia groups had similar efficacy rates and similar mortality rates (3-day efficiency rate: 68.06% vs. 63.64%, P > 0.05; 7-day efficiency rate: 76.39% vs. 71.43%, P > 0.05; mortality rate: 29.17% vs. 29.63%, P > 0.05), which illustrated the clinical value of mNGS in patients with neutropenia.

Although in our study, the prognosis of patients could be improved after adjusting the antimicrobial regimen based on mNGS, clinicians still need to be cautious in choosing mNGS testing considering that the cost of mNGS is still high (Fu et al., 2022). When suspected infections occurred in hematology patients, CMT should be chosen first, and mNGS testing can be supplemented in some circumstances, such as patients with poor therapeutic effects despite prolonged empirical use of broad-spectrum antibiotics or patients with high-risk factors that predispose them to the development of sepsis (Zhang M. et al., 2022; Feng et al., 2024). And the appropriate specimen should be selected for testing according to the site of infection. Direct specimen from the site of infection should be preferred. It has been shown that plasma mNGS was limited in its effectiveness in the diagnosis of lower respiratory tract infections and was not superior to CMT (Armstrong et al., 2019; Hill et al., 2021). mNGS was time-saving and can be used to assist in the identification of early detection of pathogenic microorganisms that are difficult to detect by CMT and time-consuming to culture. mNGS complements and corroborates conventional pathogenic diagnostic techniques, and facilitates the accurate diagnosis of infection in hematology patients.

However, there are limitations in this study. First, this study is a single-center retrospective study with a limited number of cases, and the results may have biases. We look forward to a multicenter study with a larger sample size in the future. Second, there is currently no standardized interpretation of mNGS results, and the interpretation of mNGS results in this study may be subjective. Third, only plasma was included in this study, and the diagnostic value of other types of specimens in hematology patients was not compared. We hope that more different types of specimens will be included in the future for comprehensive comparison.

## 5 Conclusions

This study demonstrated the diagnostic value of mNGS with plasma in hematology patients. mNGS can detect more microorganisms with higher positive rates than CMT, especially in patients with neutropenia. mNGS with plasma had good diagnostic value for bacterial bloodstream infections, CMV viremia, and EBV viremia. mNGS has better clinical value in patients with HSCT or neutropenia and has a positive effect on treatment and prognosis.

## Data availability statement

The original contributions presented in the study are included in the article/**Supplementary Material**. Further inquiries can be directed to the corresponding authors.

## Ethics statement

The studies involving humans were approved by the West China Hospital of Sichuan University Ethics Committee (2023-890). The studies were conducted in accordance with the local legislation and institutional requirements. Written informed consent for participation was not required from the participants or the participants’ legal guardians/next of kin in accordance with the national legislation and institutional requirements.

## Author contributions

YC: Data curation, Formal Analysis, Investigation, Methodology, Software, Writing – original draft. JW: Formal Analysis, Investigation, Methodology, Software, Writing – original draft. XG: Data curation, Formal Analysis, Investigation, Methodology, Writing – original draft. ML: Investigation, Methodology, Software, Writing – original draft. YL: Data curation, Investigation, Writing – original draft. YZ: Conceptualization, Project administration, Resources, Supervision, Validation, Visualization, Writing – review & editing. TN: Conceptualization, Funding acquisition, Project administration, Resources, Supervision, Validation, Visualization, Writing – review & editing.

## References

[B1] Chinese Society of Hematology C. M. A. Chinese Medical Doctor Association. (2020). Chinese guidelines for the clinical application of antibacterial drugs for agranulocytosis with fever (2020). Zhonghua xue ye xue za zhi= Zhonghua xueyexue zazhi 41 (12), 969–978. doi: 10.3760/cma.j.issn.0253-2727.2020.12.001 33445842 PMC7840550

[B2] Armstrong A. E. Rossoff J. Hollemon D. Hong D. K. Muller W. J. Chaudhury S. (2019). Cell-free DNA next-generation sequencing successfully detects infectious pathogens in pediatric oncology and hematopoietic stem cell transplant patients at risk for invasive fungal disease. Pediatr. Blood Cancer 66 (7), e27734. doi: 10.1002/pbc.27734 30941906

[B3] Averbuch D. Orasch C. Cordonnier C. Livermore D. M. Mikulska M. Viscoli C.. (2013). European guidelines for empirical antibacterial therapy for febrile neutropenic patients in the era of growing resistance: summary of the 2011 4th European Conference on Infections in Leukemia. Haematologica 98 (12), 1826–1835. doi: 10.3324/haematol.2013.091025 24323983 PMC3856957

[B4] Cai L. J. Wei X. L. Wei Y. Q. Guo X. T. Jiang X. J. Zhang Y.. (2023). [A single-center study on the distribution and antibiotic resistance of pathogens causing bloodstream infection in patients with hematological Malignancies]. Zhonghua Xue Ye Xue Za Zhi 44 (6), 479–483. doi: 10.3760/cma.j.issn.0253-2727.2023.06.006 37550203 PMC10450548

[B5] Daniels L. M. Durani U. Barreto J. N. O’Horo J. C. Siddiqui M. A. Park J. G.. (2019). Impact of time to antibiotic on hospital stay, intensive care unit admission, and mortality in febrile neutropenia. Support Care Cancer 27 (11), 4171–4177. doi: 10.1007/s00520-019-04701-8 30805726

[B6] Dropulic L. K. Lederman H. M. (2016). Overview of infections in the immunocompromised host. Microbiol. Spectr. 4 (4), 1–50. doi: 10.1128/microbiolspec.DMIH2-0026-2016 PMC8428766 27726779

[B7] Duan L. W. Qu J. L. Wan J. Xu Y. H. Shan Y. Wu L. X.. (2021). Effects of viral infection and microbial diversity on patients with sepsis: A retrospective study based on metagenomic next-generation sequencing. World J. Emerg. Med. 12 (1), 29–35. doi: 10.5847/wjem.j.1920-8642.2021.01.005 33505547 PMC7790710

[B8] Feng S. Rao G. Wei X. Fu R. Hou M. Song Y.. (2024). Clinical metagenomic sequencing of plasma microbial cell-free DNA for febrile neutropenia in patients with acute leukaemia. Clin. Microbiol. Infect. 30 (1), 107–113. doi: 10.1016/j.cmi.2023.05.034 37271194

[B9] Freifeld A. G. Bow E. J. Sepkowitz K. A. Boeckh M. J. Ito J. I. Mullen C. A.. (2011). Clinical practice guideline for the use of antimicrobial agents in neutropenic patients with cancer: 2010 update by the infectious diseases society of america. Clin. Infect. Dis. 52 (4), e56–e93. doi: 10.1093/cid/cir073 21258094

[B10] Fu Y. Zhu X. Cao P. Shen C. Qian X. Miao H.. (2022). Metagenomic next-generation sequencing in the diagnosis of infectious fever during myelosuppression among pediatric patients with hematological and neoplastic diseases. Infect. Drug Resist. 15, 5425–5434. doi: 10.2147/idr.S379582 36124109 PMC9482462

[B11] Govender K. N. Street T. L. Sanderson N. D. Eyre D. W. (2021). Metagenomic sequencing as a pathogen-agnostic clinical diagnostic tool for infectious diseases: a systematic review and meta-analysis of diagnostic test accuracy studies. J. Clin. Microbiol. 59 (9), e0291620. doi: 10.1128/jcm.02916-20 33910965 PMC8373000

[B12] Gu W. Deng X. Lee M. Sucu Y. D. Arevalo S. Stryke D.. (2021). Rapid pathogen detection by metagenomic next-generation sequencing of infected body fluids. Nat. Med. 27 (1), 115–124. doi: 10.1038/s41591-020-1105-z 33169017 PMC9020267

[B13] Gu W. Miller S. Chiu C. Y. (2019). Clinical metagenomic next-generation sequencing for pathogen detection. Annu. Rev. Pathol. 14, 319–338. doi: 10.1146/annurev-pathmechdis-012418-012751 30355154 PMC6345613

[B14] Guo F. Kang L. Zhang L. (2022). mNGS for identifying pathogens in febrile neutropenic children with hematological diseases. Int. J. Infect. Dis. 116, 85–90. doi: 10.1016/j.ijid.2021.12.335 34929357

[B15] Gustinetti G. Mikulska M. (2016). Bloodstream infections in neutropenic cancer patients: A practical update. Virulence 7 (3), 280–297. doi: 10.1080/21505594.2016.1156821 27002635 PMC4871679

[B16] Han D. Li Z. Li R. Tan P. Zhang R. Li J. (2019). mNGS in clinical microbiology laboratories: on the road to maturity. Crit. Rev. Microbiol. 45 (5-6), 668–685. doi: 10.1080/1040841x.2019.1681933 31691607

[B17] Hao S. F. Wang Y. H. Li L. J. Wang H. Q. Song J. Wu Y. H.. (2022). [Clinical application value of peripheral blood metagenomic next-generation sequencing test for patients with hematological diseases accompanied by fever]. Zhonghua Xue Ye Xue Za Zhi 43 (9), 766–770. doi: 10.3760/cma.j.issn.0253-2727.2022.09.009 36709171 PMC9613497

[B18] Hill J. A. Dalai S. C. Hong D. K. Ahmed A. A. Ho C. Hollemon D.. (2021). Liquid biopsy for invasive mold infections in hematopoietic cell transplant recipients with pneumonia through next-generation sequencing of microbial cell-free DNA in plasma. Clin. Infect. Dis. 73 (11), e3876–e3883. doi: 10.1093/cid/ciaa1639 33119063 PMC8664431

[B19] Huang J. Jiang E. Yang D. Wei J. Zhao M. Feng J.. (2020). Metagenomic next-generation sequencing versus traditional pathogen detection in the diagnosis of peripheral pulmonary infectious lesions. Infect. Drug Resist. 13, 567–576. doi: 10.2147/idr.S235182 32110067 PMC7036976

[B20] Lionakis M. S. Netea M. G. Holland S. M. (2014). Mendelian genetics of human susceptibility to fungal infection. Cold Spring Harb. Perspect. Med. 4 (6), 256–260. doi: 10.1101/cshperspect.a019638 PMC4031953 24890837

[B21] Liu W. Fan Z. Zhang Y. Huang F. Xu N. Xuan L.. (2021). Metagenomic next-generation sequencing for identifying pathogens in central nervous system complications after allogeneic hematopoietic stem cell transplantation. Bone Marrow Transplant. 56 (8), 1978–1983. doi: 10.1038/s41409-021-01243-8 33824437 PMC8023769

[B22] Liu W. D. Yen T. Y. Liu P. Y. Wu U. I. Bhan P. Li Y. C.. (2021). Clinical application of metagenomic next-generation sequencing in patients with hematologic Malignancies suffering from sepsis. Microorganisms 9 (11), 2309. doi: 10.3390/microorganisms9112309 34835435 PMC8624204

[B23] Miao Q. Ma Y. Wang Q. Pan J. Zhang Y. Jin W.. (2018). Microbiological diagnostic performance of metagenomic next-generation sequencing when applied to clinical practice. Clin. Infect. Dis. 67 (suppl_2), S231–s240. doi: 10.1093/cid/ciy693 30423048

[B24] Pagano L. Valentini C. G. Fianchi L. Caira M. (2009). The role of neutrophils in the development and outcome of zygomycosis in haematological patients. Clin. Microbiol. Infect. 15 (Suppl 5), 33–36. doi: 10.1111/j.1469-0691.2009.02977.x 19754754

[B25] Parize P. Muth E. Richaud C. Gratigny M. Pilmis B. Lamamy A.. (2017). Untargeted next-generation sequencing-based first-line diagnosis of infection in immunocompromised adults: a multicentre, blinded, prospective study. Clin. Microbiol. Infect. 23 (8), 574.e571–574.e576. doi: 10.1016/j.cmi.2017.02.006 28192237

[B26] Ren J. Kang J. B. Ma Y. P. Zhang J. H. Dong C. X. Kang J. M.. (2021). [Pathogen distribution and antimicrobial resistance among lower respiratory tract infections in patients with hematological Malignancies]. Zhonghua Nei Ke Za Zhi 60 (10), 875–879. doi: 10.3760/cma.j.cn112138-20201228-01056 34551475

[B27] Rolston K. V. Bodey G. P. Safdar A. (2007). Polymicrobial infection in patients with cancer: an underappreciated and underreported entity. Clin. Infect. Dis. 45 (2), 228–233. doi: 10.1086/518873 17578784

[B28] Ruan Z. Zou S. Wang Z. Zhang L. Chen H. Wu Y.. (2022). Toward accurate diagnosis and surveillance of bacterial infections using enhanced strain-level metagenomic next-generation sequencing of infected body fluids. Brief Bioinform. 23 (2), 2. doi: 10.1093/bib/bbac004 35108376

[B29] Sahu K. K. (2021). Infectious disease in hematopoietic stem cell transplantation. Ther. Adv. Infect. Dis. 8, 20499361211005600. doi: 10.1177/20499361211005600 33953915 PMC8058785

[B30] Shen Z. Wang Y. Bao A. Yang J. Sun X. Cai Y.. (2023). Metagenomic next-generation sequencing for pathogens in bronchoalveolar lavage fluid improves the survival of patients with pulmonary complications after allogeneic hematopoietic stem cell transplantation. Infect. Dis. Ther. 12 (8), 2103–2115. doi: 10.1007/s40121-023-00850-w 37541984 PMC10505113

[B31] Simner P. J. Miller S. Carroll K. C. (2018). Understanding the promises and hurdles of metagenomic next-generation sequencing as a diagnostic tool for infectious diseases. Clin. Infect. Dis. 66 (5), 778–788. doi: 10.1093/cid/cix881 29040428 PMC7108102

[B32] Sun J. H. Zhang X. H. Mo X. D. Fu H. X. Zhang Y. Y. Chen Y. Y.. (2022). Application value of metagenomic next-generation sequencing for infectious pathogens in patients receiving allogeneic hematopoietic stem cell transplantation. Zhonghua nei ke za zhi 61 (8), 928–932. doi: 10.3760/cma.j.cn112138-20220212-00104 35922218

[B33] Sun Y.-Q. Zhou J.-R. Yin Y. Zhang J.-P. Zhang M.-X. He Y.. (2022). The role of metagenomics next-generation sequencing (mNGS) in early diagnosis of bloodstream infection in hematologic patients with febrile neutropenia: A multicenter prospective study. Blood 140, 4984–4985. doi: 10.1182/blood-2022-165036

[B34] Valdez J. M. Scheinberg P. Nunez O. Wu C. O. Young N. S. Walsh T. J. (2011). Decreased infection-related mortality and improved survival in severe aplastic anemia in the past two decades. Clin. Infect. Dis. 52 (6), 726–735. doi: 10.1093/cid/ciq245 21367725 PMC3106262

[B35] Xu C. Chen X. Zhu G. Yi H. Chen S. Yu Y.. (2022). Utility of plasma cell-free DNA next-generation sequencing for diagnosis of infectious diseases in patients with hematological disorders. J. Infection 86 (1), 14–23. doi: 10.1016/j.jinf.2022.11.020 36462587

[B36] Yang L. Song J. Wang Y. Feng J. (2021). Metagenomic next-generation sequencing for pulmonary fungal infection diagnosis: lung biopsy versus bronchoalveolar lavage fluid. Infect. Drug Resist. 14, 4333–4359. doi: 10.2147/idr.S333818 34707378 PMC8542593

[B37] Zhang B. Gui R. Wang Q. Jiao X. Li Z. Wang J.. (2022). Comparing the application of mNGS after combined pneumonia in hematologic patients receiving hematopoietic stem cell transplantation and chemotherapy: A retrospective analysis. Front. Cell. Infection Microbiol. 12. doi: 10.3389/fcimb.2022.969126 PMC9532739 36211959

[B38] Zhang X. Li Y. Yin J. Xi B. Wang N. Zhang Y. (2022). Application of next-generation sequencing in infections after allogeneic haematopoietic stem cell transplantation: A retrospective study. Front. Cell. Infection Microbiol. 12. doi: 10.3389/fcimb.2022.888398 PMC9239075 35774403

[B39] Zhang M. Wang Z. Wang J. Lv H. Xiao X. Lu W.. (2022). The value of metagenomic next-generation sequencing in hematological Malignancy patients with febrile neutropenia after empiric antibiotic treatment failure. Infection Drug Resistance 15, 3549–3559. doi: 10.2147/idr.S364525 35837537 PMC9273631

[B40] Zhang X. Wang F. Yu J. Jiang Z. (2023). Clinical application value of metagenomic second-generation sequencing technology in hematologic diseases with and without transplantation. Front. Cell. Infection Microbiol. 13. doi: 10.3389/fcimb.2023.1135460 PMC10311908 37396304

[B41] Zhang P. Zhang Z. H. Liang J. Shen D. Y. Li J. Wang D.. (2022). Metagenomic next-generation sequencing for the diagnosis of fever of unknown origin in pediatric patients with hematological Malignancy. Clinica Chimica Acta 537, 133–139. doi: 10.1016/j.cca.2022.10.008 36283493

[B42] Zhong S. Yang M.-H. (2023). Value of metagenomic next-generation sequencing in children with hematological Malignancies complicated with infections. Zhongguo dang dai er ke za zhi = Chin. J. Contemp. Pediatr. 25 (7), 718–725. doi: 10.7499/j.issn.1008-8830.2212059 PMC10414172 37529954

