# Supplementary material for: Application of plasma metagenomic next-generation sequencing improves prognosis in hematology patients with neutropenia or hematopoietic stem cell transplantation for infection
Source: Front Cell Infect Microbiol. 2024 Feb 2;14:1338307. doi: 10.3389/fcimb.2024.1338307 (PMC10869557; doi:10.3389/fcimb.2024.1338307)
Supplement: Supplementary file 1 [file Table_1.docx]

Supplementary Material

Application of Plasma Metagenomic Next-generation Sequencing Improves Prognosis in Hematology Patients with Neutropenia or Hematopoietic Stem Cell Transplantation for Infection

Yuhui Chen ^1†^, Jinjin Wang ^1†^, Xinai Gan ^1^, Meng Li ^1^, Yi Liao ^1^, Yongzhao Zhou ^2*^ and Ting Niu^1*^

^†^These authors contributed equally to this work and share first authorship

^1^ Department of Hematology, West China Hospital, Sichuan University, Chengdu, China

^2^ Integrated Care Management Center, West China Hospital, Sichuan University, Chengdu, China

*** Correspondence:
Ting Niu**

Department of Hematology, West China Hospital, Sichuan University, Chengdu, China

Email: niuting@wchscu.cn

Postal address: No. 37 GuoXueXiang Street，, Chengdu, Sichuan Province 610041, China.

**Yongzhao Zhou**

Integrated Care Management Center, West China Hospital, Sichuan University, Chengdu, Chinar

Email: [yongzhaozhou001@wchscu.cn](mailto:yongzhaozhou001@wchscu.cn)

Postal address: No. 37 GuoXueXiang Street, Chengdu, Sichuan Province 610041, China.

Table S1 Detection positive rates of pathogen in HSCT and non-HSCT groups

| Pathogen | Group | Positive Rate | P Value |
| --- | --- | --- | --- |
| Total | HSCT | 84.44% | 0.259 |
|  | Non-HSCT | 90.74% |  |
| Bacteria | HSCT | 33.3% | 0.664 |
|  | Non-HSCT | 37.0% |  |
| Virus | HSCT | 20.0% | 0.959 |
|  | Non-HSCT | 20.4% |  |
| Fungi | HSCT | 62.2% | 0.179 |
|  | Non-HSCT | 73.1% |  |

Table S2 Comparison of Adjustment and non- Adjustment groups

|  | Hospitalization Day, Median (quartile) | Time of  Neutropenia, Median (quartile) | CRP, Median (quartile) | PCT, Median (quartile) | IL-6, Median (quartile) |
| --- | --- | --- | --- | --- | --- |
| Adjustment | 50(35-73.5) | 7 (0-20) | 95(44.9-154) | 0.47(0.27-1.46) | 112.2(39.8-258.5) |
| Non- adjustment | 43(28-72) | 0(0-7) | 69.39(18-118) | 0.505(0.17-1.55) | 71.85(17.9-226.3) |
| P Value | 0.275 | **0.002** | **0.048** | 0.684 | 0.161 |

Table S3 Comparison of HSCT and non- HSCT groups

|  | Time of  Neutropenia, Median (quartile) | CRP, Median (quartile) | PCT, Median (quartile) | IL-6, Median (quartile) |
| --- | --- | --- | --- | --- |
| HSCT | 2(0-7) | 73.9(17.9-145) | 0.61(0.34-2.52) | 65.9(16.4-296) |
| Non- HSCT | 0(0-13.5) | 76.25(23.45-137) | 0.43(0.18-1.16) | 106.1(31.7-208) |
| P Value | 0.842 | 0.992 | 0.124 | 0.596 |
